# The effect of a dynamic lighting schedule on neurobehavioral performance during a 45-day simulated space mission

**DOI:** 10.1093/sleepadvances/zpae032

**Published:** 2024-05-30

**Authors:** Leilah K Grant, Brianne A Kent, Shadab A Rahman, Melissa A St. Hilaire, Crystal L Kirkley, Kevin B Gregory, Toni Clark, John P Hanifin, Laura K Barger, Charles A Czeisler, George C Brainard, Steven W Lockley, Erin E Flynn-Evans

**Affiliations:** Division of Sleep and Circadian Disorders, Brigham & Women’s Hospital, Boston, MA, USA; Division of Sleep Medicine, Harvard Medical School, Boston, MA, USA; Division of Sleep and Circadian Disorders, Brigham & Women’s Hospital, Boston, MA, USA; Division of Sleep Medicine, Harvard Medical School, Boston, MA, USA; Division of Sleep and Circadian Disorders, Brigham & Women’s Hospital, Boston, MA, USA; Division of Sleep Medicine, Harvard Medical School, Boston, MA, USA; Division of Sleep and Circadian Disorders, Brigham & Women’s Hospital, Boston, MA, USA; Division of Sleep Medicine, Harvard Medical School, Boston, MA, USA; Fatigue Countermeasures Laboratory, Human Systems Integration Division, NASA Ames Research Center, Moffett Field, CA, USA; Fatigue Countermeasures Laboratory, Human Systems Integration Division, NASA Ames Research Center, Moffett Field, CA, USA; Johnson Space Center, Houston, TX, USA; Department of Neurology, Thomas Jefferson University, Philadelphia, PA, USA; Division of Sleep and Circadian Disorders, Brigham & Women’s Hospital, Boston, MA, USA; Division of Sleep Medicine, Harvard Medical School, Boston, MA, USA; Division of Sleep and Circadian Disorders, Brigham & Women’s Hospital, Boston, MA, USA; Division of Sleep Medicine, Harvard Medical School, Boston, MA, USA; Department of Neurology, Thomas Jefferson University, Philadelphia, PA, USA; Division of Sleep and Circadian Disorders, Brigham & Women’s Hospital, Boston, MA, USA; Division of Sleep Medicine, Harvard Medical School, Boston, MA, USA; Fatigue Countermeasures Laboratory, Human Systems Integration Division, NASA Ames Research Center, Moffett Field, CA, USA

**Keywords:** psychomotor vigilance task, sleep restriction, spaceflight, neurobehavioral performance, chronic variable sleep restriction, light, dynamic lighting, circadian

## Abstract

**Study Objectives:**

We previously reported that during a 45-day simulated space mission, a dynamic lighting schedule (DLS) improved circadian phase alignment and performance assessed once on selected days. This study aimed to evaluate how DLS affected performance on a 5-minute psychomotor vigilance task (PVT) administered multiple times per day on selected days.

**Methods:**

Sixteen crewmembers (37.4 ± 6.7 years; 5F) underwent six cycles of 2 × 8-hour/night followed by 5 × 5-hour/night sleep opportunities. During the DLS (*n* = 8), daytime white light exposure was blue-enriched (~6000 K; Level 1: 1079, Level 2: 76 melanopic equivalent daytime illuminance (melEDI) lux) and blue-depleted (~3000–4000 K; L1: 21, L2: 2 melEDI lux) 3 hours before bed. In the standard lighting schedule (SLS; *n* = 8), lighting remained constant (~4500K; L1: 284, L2 62 melEDI lux). Effects of lighting condition (DLS/SLS), sleep condition (5/8 hours), time into mission, and their interactions, and time awake on PVT performance were analyzed using generalized linear mixed models.

**Results:**

The DLS was associated with fewer attentional lapses (reaction time [RT] > 500 milliseconds) compared to SLS. Lapses, mean RT, and 10% fastest/slowest RTs were worse following 5 compared to 8 hours of sleep but not between lighting conditions. There was an effect of time into mission on RTs, likely due to sleep loss. Overall performance differed by time of day, with longer RTs at the beginning and end of the day. There were more lapses and slower RTs in the afternoon in the SLS compared to the DLS condition.

**Conclusions:**

Future missions should incorporate DLS to enhance circadian alignment and performance. This paper is part of the *Sleep and Circadian Rhythms: Management of Fatigue in Occupational Settings* Collection.

**Statement of Significance**

Chronic sleep restriction is common during spaceflight leaving astronauts vulnerable to neurobehavioral performance impairment. A dynamic lighting schedule (DLS) with alertness-promoting high illuminance, blue-enriched white light during the day, and sleep-promoting dim, blue-depleted white light in the evening is a promising non-pharmacological countermeasure for sleep loss-related performance impairment. Therefore, we tested the effects of a DLS compared to a standard static lighting schedule on neurobehavioral performance assessed multiple times per day on selected days during a 45-day simulated space mission. We found that the DLS was protective against the cumulative decline in performance due to chronic sleep restriction suggesting that future space missions may benefit from the incorporation of dynamic lighting.

Developing countermeasures to mitigate safety, performance, and health impairments associated with circadian misalignment is particularly important for spaceflight, during which crewmembers are often exposed to unusual light–dark and work schedules. Circadian misalignment can occur because of insufficient or inappropriately timed light exposure, or because work is scheduled during the biological night to accommodate operational needs. Circadian misalignment has been identified during most spaceflight and analog missions studied to date [1–9], increasing the risk of reduced sleep quality and impaired cognition.

In addition to circadian misalignment, chronic sleep restriction is also common during spaceflight. It has been consistently reported that astronauts sleep on average only ~6 hour/night (reviewed by [10]), which is less than the recommended minimum of 7 hours for adults to promote optimal alertness and health [11] and is a sleep duration that is associated with impaired performance [12, 13]. Under operational conditions, sleep patterns tend to be more variable, with repeated cycles of chronic sleep loss and short-term recovery (e.g. weekdays vs. weekends). Such chronic variable sleep loss exacerbates performance impairment more than simple sleep duration predicts [14]. In a recent study, we showed that while performance appeared to be restored after 1 night of 10-hour recovery sleep, when re-challenged with a second or third cycle of chronic sleep loss, performance remained impaired and deteriorated at a faster rate [15]. Similarly, another laboratory study simulating one work week with restricted sleep (6 hours/night) and a weekend with extended recovery sleep (10 hours/night), found that performance did not improve to baseline levels after the recovery sleep, despite improvement in subjective and objective measurements of sleepiness [14]. Devising countermeasures to tackle these more realistic sleep patterns is required to improve operational health and safety.

Light exposure is a promising non-pharmacological countermeasure for sleepiness because light has both circadian resetting [16] and direct alerting effects [17]. The National Aeronautics and Space Administration (NASA) has installed a new solid-state lighting assembly (SSLA) on the International Space Station (ISS), which permits variation in the white light spectrum and illuminance depending on the operational needs [18]. Using the SSLA, we have deployed a dynamic lighting schedule (DLS) incorporating episodes of white light for general vision, blue-enriched high-illuminance white light for enhanced alertness and circadian adaptation [18], and blue-depleted lower illuminance white light to reduce alertness prior to sleep [19].

The present study compared DLS to a standard lighting schedule (SLS) during 45-day missions in the Human Exploration Research Analog (HERA) habitat (Campaign 4) (https://www.nasa.gov/mission/hera/). We previously reported that misalignment of the circadian rhythm of 6-sulphatoxymelatonin (aMT6s), the urinary metabolite of melatonin that is a reliable marker of the circadian phase, was improved under the DLS condition, compared to standard lighting [20]. The effects of light on performance, measured once per day in the evening on selected days throughout the mission using the Cognition battery [21, 22], were less clear with improvements in some, but not all measures [20]. The assessment of performance was also obscured by having approximately half of the once-per-day tests scheduled during the wake maintenance zone (WMZ), a time at which the circadian system promotes arousal, making it harder to identify performance impairment [23–26]. To address this shortcoming, we analyzed performance measured five times per day on selected days during the mission to assess the change in the time course of performance over the day during extended exposure to chronic variable sleep loss, and also examined the impact of the DLS on attenuating any performance impairment across the mission. Performance was assessed using a 5-minute Psychomotor Vigilance Task (PVT), which is a well-validated measure of sustained visual attention that is sensitive to acute sleep deprivation and chronic sleep restriction [12, 13, 27, 28], as well as circadian phase [1, 29–31].

## Materials and Methods

Detailed methods were previously published in Rahman et al. [20] and Flynn-Evans et al. [32] and are summarized below. Using data from the same crewmembers, the effects of the lighting schedules on circadian phase, sleep, and performance on the Cognition battery (performed only once per day in the evening on selected days) were reported by Rahman et al. [20] and the effects of the chronic variable sleep schedule, but not lighting condition, on the 5-minute PVT were reported by Flynn-Evans et al. [32]. The current analysis described the effects of the lighting schedule (SLS vs. DLS) on the 5-minute PVT performed multiple times across the day, which has not previously been reported.

### Participants

Participants were selected to be “astronaut like” (i.e. met the NASA long-duration space flight physical standards, were in astronaut age range and educational background as described in [32]) and included 20 (7 female) healthy adults 30–55 years of age with at least college-level education. All participants provided written informed consent prior to the study. All study procedures conformed to the guidelines set forth in the United States Common Rule. This study was approved by the NASA Johnson Space Center Institutional Review Board (protocol PRO2328) and Partners Healthcare (protocol 2017P000059).

### Human exploration research analog habitat

The HERA habitat is a three-story unit that has an airlock, a hygiene module, and crew quarters (Supplementary Figure S1). The habitat contains spaceflight simulation workstations, a galley, a communication station, an aerobic exercise station, and private sleep quarters. The habitat room temperature was maintained at 72° F (± 5° F), with 70% (± 10%) humidity for all missions.

### Mission schedule and procedures

**Figure 1.**
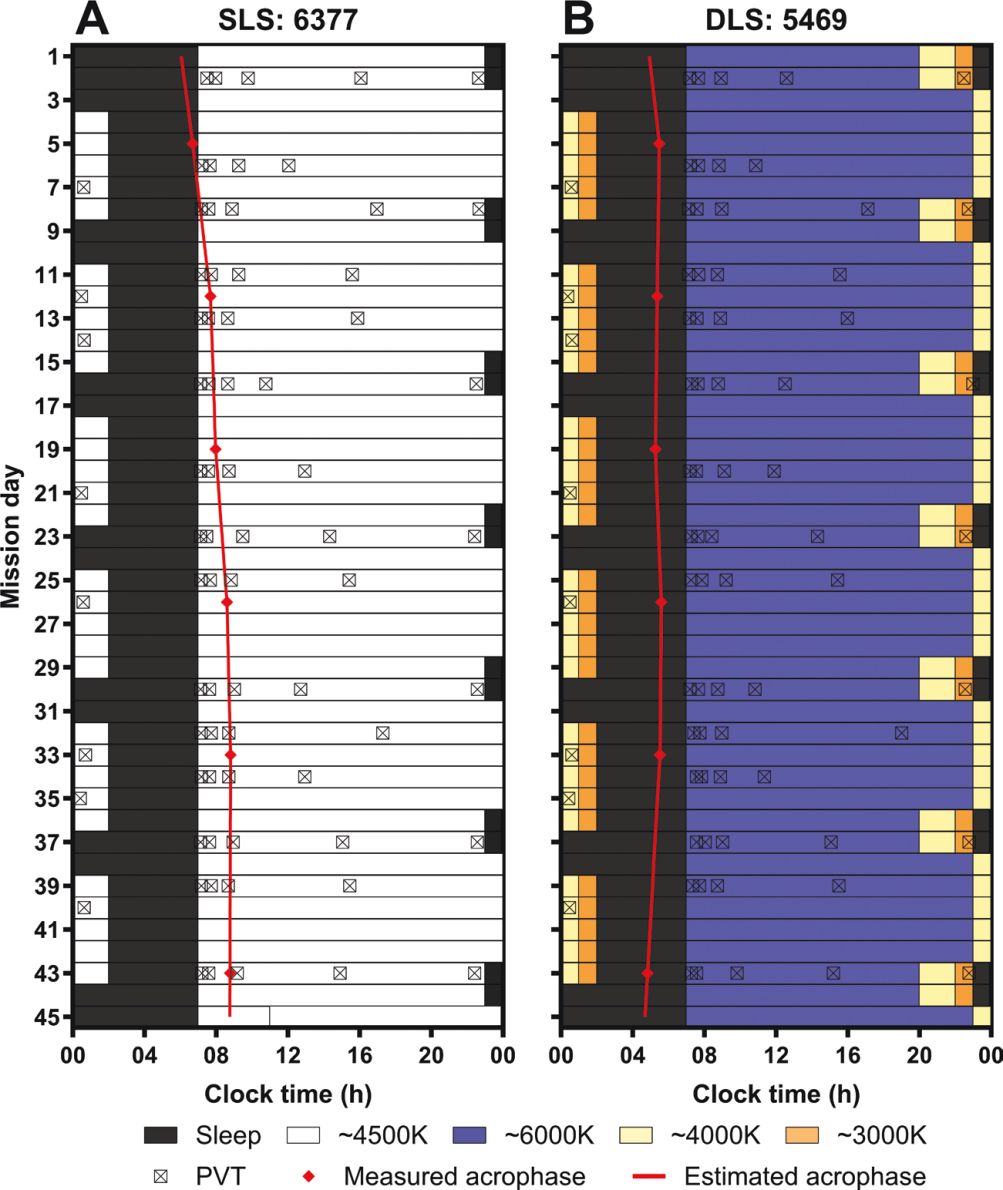
Representative study protocol plotted in raster format. (A) crewmember 6377 studied under the standard lighting schedule (SLS) and (B) crewmember 5469 studied under the dynamic lighting schedule (DLS). The *y*-axis indicates the mission day and the *x*-axis indicates the clock time. The black bars represent the scheduled sleep period. The white bars represent wake under standard lighting (~4500 K) and the blue bars represent wake under bright blue-enriched light (~6000 K). The yellow and orange bars represent when the lighting was reduced in intensity and short-wavelength content (~4000 and ~3000 K, respectively). The measured aMT6s acrophase is indicated by the red diamonds and the estimated daily acrophase of aMT6s is indicated by the red line. The timing of the PVT testing is indicated by squares marked with an x.

Five missions were scheduled for HERA Campaign 4. Each mission was scheduled for 45 days and followed the same study schedule. Following an initial 8-hour sleep opportunity, crewmembers were scheduled to sleep for 6 cycles of 8 hours/night for two consecutive nights followed by 5 hours/night for 5 nights, ending with 2 × 8 hours/night recovery sleep episodes, aligned by waketime (Figure 1). Napping outside of scheduled sleep episodes was not permitted. Caffeine (maximum two cups) was only allowed between 07:45 and 14:00 hours. The precise amount and timing of caffeine consumption were not recorded.

Crewmembers were scheduled to complete a 5-minute PVT five times a day on 15 mission days, every 2–5 days throughout the mission (Figure 1). The five PVT sessions were scheduled: (1) within 30 minutes of waking, (2) 30 minutes after session 1, (3) mid-morning, (4) mid-afternoon, and (5) before going to bed. The exact timing of the five tests were variable between crewmembers and across mission days (Figure 1). The 15 testing days were the same for all missions and were scheduled according to availability relative to the simulated operational tasks (e.g. extravehicular activities, robotic arm maneuvres, and science experiments) that were conducted by the crewmembers throughout the mission.

The version of the PVT used was the NASA-PVT, a previously validated touchscreen version of the 5-minute PVT performed on a fifth-generation, 32-GB Apple iPod (Apple Inc. Cupertino, CA) running IOS 9.3.5 [33]. The visual stimulus was a 3-mm reaction time (RT) counter with an interstimulus interval of 2 to 10 seconds, and a timeout of 10 seconds. The test was administered with the device held in landscape orientation. Participants responded to the stimuli by using the thumb of their dominant hand to press either the left or right side of the screen.

### Lighting conditions

The SLS was deployed throughout missions 1 and 2, and the DLS throughout missions 3, 4, and 5. Overall, the DLS resulted in higher melanopic EDI (Equivalent Daylight Illuminance) and photopic (visual) lux [34] exposure in the day, and lower levels during the evening, as compared to the SLS condition (see extensive details provided in Table 1 in Rahman et al. [20],). In brief, when measured at a height of 72″ in the horizontal plane on level 1, the main daytime working area, the DLS increased illuminance during the day (1079 melEDI lux and 1210 photopic lux), compared to the SLS condition (284 melEDI lux and 467 photopic lux). On level 2 where most time was spent during the evening, the DLS condition substantially reduced the illuminance (2 melEDI lux, 4 photopic lux,) compared to the SLS condition (62 melEDI lux, 108 photopic lux). The bunk light illuminance, measured in the vertical plane from the center of the bunk space, was also reduced from 101 melEDI lux and 259 photopic lux during the SLS condition to 21 melEDI lux and 63 photopic lux during the DLS condition without substantial changes in the light spectrum.

Although the data were collected prior to their publication, the melEDI lux of the level 1 daytime (52″ vertical 233 ± 119 melEDI lux) and level 2 evening and night (52″ vertical 3 ± 0, and 2 ± 0 melEDI lux, respectively) settings in the DLS condition are broadly consistent with 250, 10, and 1 melEDI lux thresholds suggested for the daytime, evening and nighttime light exposures, respectively, in recently published guidelines. The SLS condition, however, did not meet the melEDI lux thresholds recommended by the guidelines [35]. While the bunk space did not meet these guidelines due to the geometry of the light within the confined space of the bunks, the melEDI lux was substantially reduced in the DLS compared to the SLS condition as described above.

### Urine collection

Serial urine samples were collected from each crewmember over 48 hours per week and assayed for 6-sulphatoxymelatonin (aMT6s; Surrey Assays, UK), to assess changes in circadian phase during the study [20].

### Data analysis

For aMT6s, 2.3% (53/2231) samples were removed from the analysis due to missing sample information (e.g. collection time or volume) or values being outside of normative ranges. Of the 146 48-hour profiles analyzed, all but one (*p *= .06, removed from analyses) had a statistically significant (*p *< .05) cosinor rhythm. See Rahman et al. [20] for further details. The average acrophase across the mission was compared between groups using the Wilcoxon two-sample test.

Four PVT outcomes were considered in overall analyses: (1) lapses of attention [RT > 500 milliseconds], (2) mean RT, (3) mean RT of 10% fastest responses, and (4) mean RT of 10% slowest responses. These outcomes have previously been shown to be associated with sleep loss and impaired performance [36] and are sensitive to the alerting effects of light [37–39]. To examine the effects of lighting condition (SLS vs. DLS), sleep condition (5 vs. 8 hours), time into the mission (i.e. mission tertile), and their interactions, individual-level PVT data from repeated daily tests were averaged within each light and sleep condition across three 2-week blocks and were analyzed using generalized linear mixed models (GLMM) with participant-level random effects and a negative binomial (lapses) or log-normal (RT outcomes) distribution. To examine the effects of time awake and the interaction between time awake and lighting condition, the 5 PVT sessions were binned in 2-hour increments starting from 0 hours awake and analyzed using GLMM as described above. Due to the scheduled timing of the tests, bins centered at 11 and 13 hours since wake had <50% of participants contributing data and were therefore excluded from the analysis. Due to differences in acrophase between the lighting schedules as described below, acrophase was included as a covariate in all analyses of the neurobehavioral performance. Where statistically significant interactions were found, post hoc analyses were conducted to examine which conditions and timepoints were different using t or F tests as appropriate. Normality of residuals was assessed by visual inspection of histograms and Q-Q plots. All analyses and graphical representations were conducted in SAS 9.4 (SAS Inc., Cary, NC, USA) and GraphPad Prism 10.0.2. (GraphPad Software La Jolla, CA, USA).

## Results

Four participants (1 female) did not complete the full mission, because mission 2 (SLS) was aborted on mission day 22 due to a hurricane. As reported previously [20], mission 3 did not receive the scheduled DLS correctly and those data were therefore removed from further analyses, but the 5-minute PVT results, regardless of lighting condition, have been reported on previously [32]. Therefore, the final analysis was conducted on the 16 crewmembers from missions 1, 2, 4, and 5, including *n* = 8 exposed to SLS lighting (missions 1 and 2; mean age ± SD = 39.5 ± 7.0, range 30–50 years) and *n* = 8 exposed to DLS lighting (missions 4 and 5; mean age ± SD = 35.4 ± 6.1, range 29–48 years).

### Effects of lighting condition on acrophase

As reported previously [20], the aMT6s acrophase of crewmembers under the SLS lighting (missions 1 and 2) was, on average, 1.5 hours later (*p* = .003) than that of crewmembers under the DLS lighting (missions 4 and 5; Supplementary Figure S2). Acrophase was therefore included as a covariate in all analyses of the neurobehavioral performance data due to the well-documented effects of circadian phase on neurobehavioral performance [23, 26].

### Effects of lighting, sleep condition, and time into the mission on PVT performance

**Figure 2.**
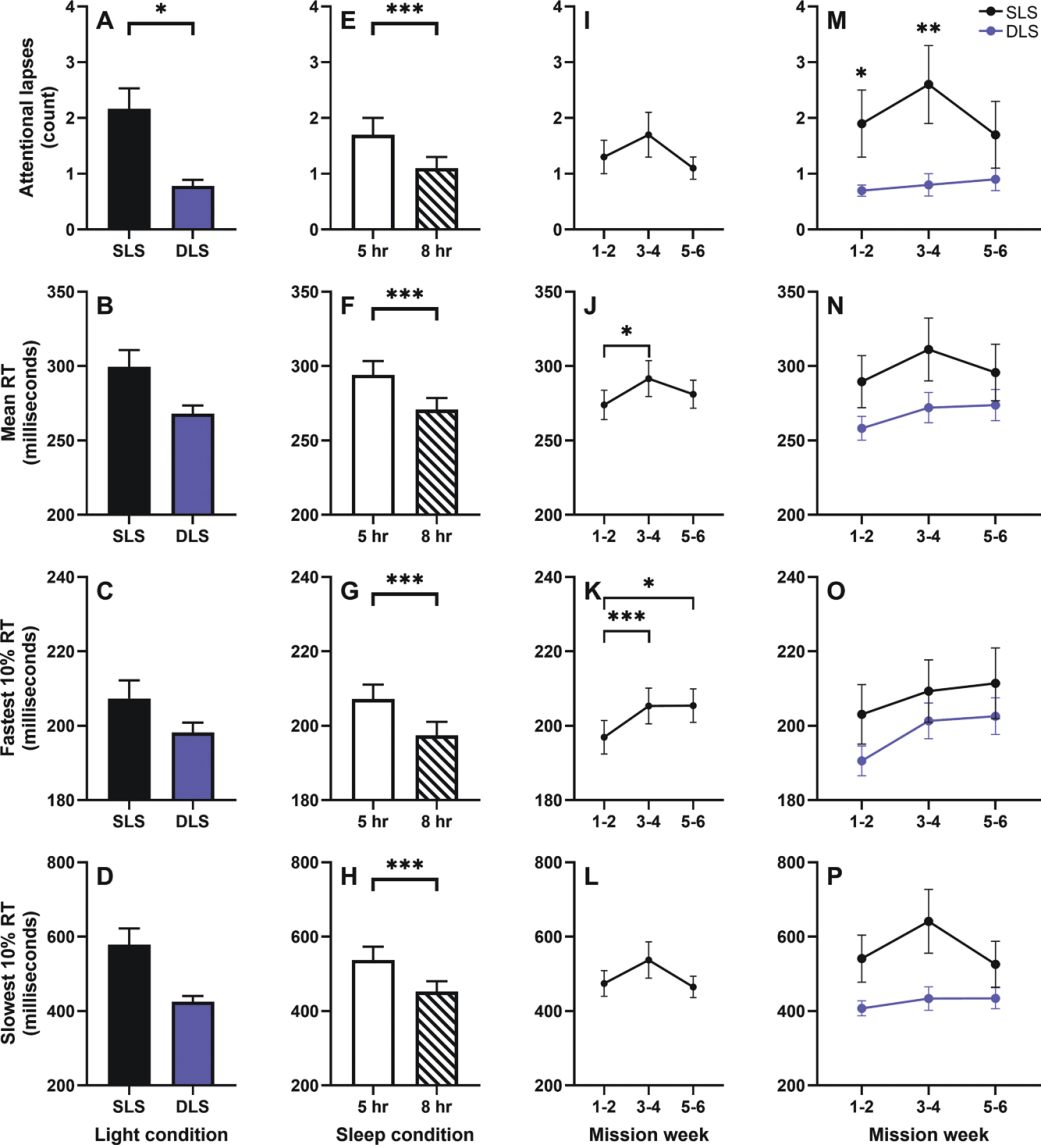
Effects of light, sleep, and time into the mission on Psychomotor Vigilance Task (PVT) performance in 16 crewmembers (*n* = 8/lighting condition). Mean ± SEM of attentional lapses (top), mean reaction time (RT; center-top), 10% fastest RT (center-bottom), and 10% slowest RT (bottom) are shown by lighting condition (A–D), sleep condition (E–H), mission week tertile (I–L) and lighting condition by mission week tertile M–P). Unadjusted data are plotted. DLS, dynamic lighting schedule; SLS, standard lighting schedule; * *p* < .05, ** *p* < .01, *** *p* < .001.

Across the whole mission, attentional lapses were significantly higher in the SLS compared to DLS lighting condition (F_1,59_ = 4.5, *p* = .04; Figure 2A). Mean RT and the 10% fastest and slowest RTs were not different between the lighting conditions, however (Figure 2B–D).

In PVT sessions following 8 compared to 5 hours of sleep, there were significantly fewer attentional lapses (F_1,59_ = 28.5, *p* < .001), and significantly faster mean RT (F_1,59_ = 31.5, *p* < .001) and 10% fastest (F_1,59_ = 34.7, *p* < .001) and slowest (F_1,59_ = 19.5, *p* < .001) RTs (Figure 2E–H).

As the time into the mission increased performance deteriorated, such that mean RT (F_2,59_ = 3.3, *p* = .04) was slower in the second tertile (t_59_ = −2.5, *p* = .01, t_59_ = −2.5, *p* = .01) and the 10% fastest RT (F_2,59_ = 7.3, *p* = .002) was slower in the second (t_59_ = −3.8, *p* < .001) and third tertiles (t_59_ = −2.1, *p* = .04) compared to the first tertile (Figure 2J and K). Attentional lapses and the 10% slowest RT did not change across the mission (Figure 2I and L).

There was a significant interaction between lighting condition and mission tertile for the fastest 10% RT (F_2,59_ = 3.2, *p* = .049) and a statistical trend for attentional lapses (F_2,59_ = 2.97, *p* = .059), but not for mean RT or the 10% slowest RT (Figure 2M–P). Post hoc analyses did not reach statistical significance for the 10% fastest RT. For attentional lapses, however, the SLS group had significantly more attentional lapses than the DLS group during the first (F_1,59_ = 4.03, *p* = .049) and second tertiles (F_1,59_ = 7.57, *p* = .008; Figure 2M).

There was a significant interaction between sleep condition and mission tertile for mean RT (F_2,59_ = 7.9, *p* < .001) and the 10% fastest (F_2,59_ = 9.8, *p* < .001) and slowest RTs (F_2,59_ = 5.8, *p* = .005), but not attentional lapses (Supplementary Figure S2A–D). When testing was performed on mission days following 5 compared to 8 hours of sleep, the mean and 10% fastest RTs were significantly slower during the first (F_1,59_ = 6.9, *p* = .01 and F_1,59_ = 10.1, *p* = .002, respectively), second (F_1,59_ = 36.3, *p* < .001 and F_1,59_ = 72.0, *p* < .001, respectively) and third tertiles (F_1,59_ = 18.5, *p* < .001 and F_1,59_ = 23.9, *p* < .001, respectively), while the 10% slowest RT was significantly slower only during the second (F_1,59_ = 18.9, *p* < .001) and third tertiles (F_1,59_ = 6.6, *p* = .01)

The interaction between sleep condition and mission tertile (Supplementary Figure S2E–H), and the three-way interaction between light condition, sleep condition, and mission tertile (Supplementary Figure S2I–L) were not significant for any of the PVT outcomes.

### Effects of time awake on PVT performance

**Figure 3.**
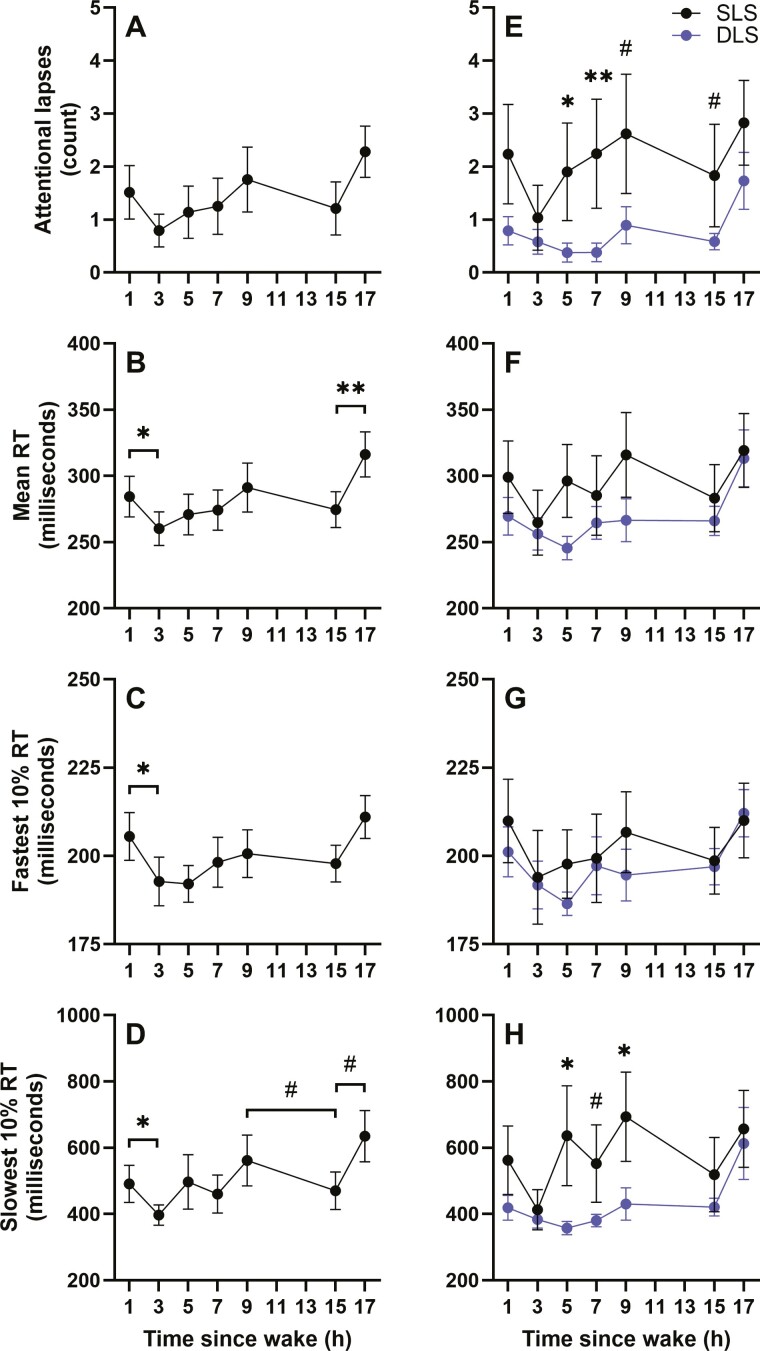
Effects of time awake on psychomotor vigilance task (PVT) performance in 16 crewmembers (*n* = 8 / lighting condition). Mean ± SEM of attentional lapses (top), mean reaction time (RT; center-top), 10% fastest RT (center-bottom), and 10% slowest RT (bottom) are shown by time awake (A–D) and time awake by lighting condition (E–H). Unadjusted data are plotted. DLS, dynamic lighting schedule; SLS, standard lighting schedule; ^#^*p* < .1 (statistical trend), * *p* < .05, ** *p* < .01.

Attentional lapses (F_6,81_ = 11.4, *p* < .001), mean RT (F_6,81_ = 10.7, *p* < .001), and the 10% fastest (F_6,81_ = 11.4, *p* < .001) and slowest RTs (F_6,81_ = 9.6, *p* < .001) differed by time awake (Figure 3A-D). Mean RT and the 10% fastest RTs were faster at 3 hours compared to 1 hour awake (t_81_ = 2.1, *p* = .04 and t_81_ = 2.3, *p* = .02, respectively), and at 15 compared to 17 hours awake (t_81_ = −3.3, *p* = .002 and t_81_ = −3.0, *p* = .003, respectively). The slowest 10% RT was faster at 3 hours compared to 1 hour awake (t_81_ = 3.0, *p* = .003) and trended toward being faster at 15 compared to 9 (t_81_ = 1.8, *p* = .08) and 15 compared to 17 (t_81_ = −1.9, *p* = .06) hours awake. Post hoc comparisons between the different time awake bins were not significantly different for attentional lapses.

There was a significant interaction between lighting condition and time awake for attentional lapses (F_6,81_ = 3.3, *p* = .006), mean RT (F_6,81_ = 4.6, *p* < .001) and the 10% fastest (F_6,81_ = 3.7, *p* = .003) and slowest RTs (F_6,81_ = 3.2, *p* = .007; Figure 3E–H). Compared to the DLS condition, the SLS lighting condition had significantly more attentional lapses after 5 (F_1,81_ = 6.1, *p* = .02) and 7 (F_1,81_ = 7.6, *p* = .007) hours awake, and trended toward more lapses after 9 (F_1,81_ = 3.5, *p* = .07) and 15 (F_1,81_ = 3.73, *p* = .06) hours awake, while the 10% slowest RT was significantly slower after 5 (F_1,81_ = 4.5, *p* = .04) and 9 (F_1,81_ = 4.1, *p* = .045) hours awake, and trended toward being slower after 7 (F_1,81_ = 3.0, *p* = .09) hours awake. Post hoc comparisons between the lighting conditions at each time awake bin were not significantly different for the mean and 10% fastest RTs.

## Discussion

Developing countermeasures for the circadian misalignment, sleep loss, and cognitive deficits associated with spaceflight are essential to the health and safety of crewmembers. Analog missions represent a highly controlled environment suitable for evaluating the effects of countermeasures on performance, including the effects of light. Here we show that during the 45-day NASA HERA4 analog mission that exposed the crewmembers to chronic variable sleep loss, a DLS incorporating periods of blue-enriched high illuminance white light during the wake episodes and blue-depleted lower illuminance light for 3 hours prior to sleep [19, 35], was associated with superior performance on the PVT compared to SLS, which kept lighting constant during waking hours.

The HERA4 mission was designed to mimic the type of chronic variable sleep loss that often occurs, not only during spaceflight, but also in many other real-world operational settings. Although there was an acute effect of prior sleep duration such that performance was worse following 5 compared to 8 hours of sleep, as expected, and confirming the findings of Flynn-Evans et al. [32], there was also chronic decline associated with the variable sleep schedule as illustrated by an increase in the mean and the fastest 10% RTs across the 2-week blocks of the mission (i.e. mission tertiles). The chronic decline in performance across the mission suggests that the 8-hour recovery sleep opportunities on weekends were not sufficient to restore performance following subsequent rechallenge with restricted 5-hour sleep opportunities during weekdays, as shown in a laboratory study of chronic variable sleep loss [15].

Light exposure is considered a circadian and sleepiness countermeasure [16–18] and solid-state lighting has been installed in the ISS for this purpose, with three settings: (1) white light, (2) blue-enriched white light to enhance circadian adaptation and alertness, and (3) blue-depleted white light to minimize alertness prior to sleep [18]. In the present study, we simulated a similar DLS to examine its potential benefits in counteracting the performance decline due to chronic variable sleep loss. As we previously reported that the DLS, compared to SLS, improved circadian adaptation [20], to isolate the effects of the lighting condition on performance impairment due to chronic variable sleep loss, all analyses included circadian phase as a covariate. When not accounting for lighting conditions or circadian phase, Flynn-Evans et al. [32] previously reported that mean RT and fastest 10% RT declined across the mission. We replicated this finding in the current analysis when examining the effect of mission tertile on performance regardless of lighting condition, but while controlling for circadian phase. When examining the interaction between mission tertile and lighting condition while controlling for circadian phase; however, we found that, while attentional lapses increased in SLS condition during the first two mission tertiles, performance stayed relatively stable and was therefore significantly better in the DLS condition (Figure 2M) suggesting that DLS was protective against the chronic decline in daytime performance resulting from the repeated exposure to chronic variable sleep restriction.

In addition to examining the effects of the sleep and lighting schedules on performance across the mission, we also examined the time course of performance during the day. Compared to tests performed in the 2 to 4 hours after waking, performance on tests within the first 2 hours of waking was worse, consistent with performance deficits related to sleep inertia [40, 41]. Additionally, performance at the end of the day was significantly better at 14 to 16 hours awake compared to both 10 to 12 hours awake (10% slowest RT) and 16 to 18 hours awake (mean and 10% slowest RTs). This improvement in performance between 14 and 16 hours awake is likely the effect of the WMZ, a time where performance is temporarily elevated due to circadian drive for alertness [24, 25]. Although the WMZ typically occurs a few hours earlier in individuals with normal circadian phase angles, a delay in the WMZ relative to time awake would be consistent with the delayed circadian phase observed in this dataset, particularly in the SLS group. These changes in performance across the day confirm that performance, even in highly motivated, highly trained professionals, is controlled by both circadian phase and time awake in operational and real-world environments [31, 42–47]

Testing multiple times across a day, rather than a single time point, is necessary to adequately evaluate performance and the efficacy of countermeasures for performance given the inability to place any single time point measure in the context of circadian phase and time awake. These two processes (Process C and S, respectively [48]) are the fundamental regulators of waking performance and interact to determine the time course of performance across a waking episode [23, 26]. Assessment of performance and the impact of countermeasures therefore have to target performance improvements in the context of these processes. This is powerfully demonstrated by comparing the performance profiles and lighting effects in our previous paper [20], when performance was measured once in the evening, with the current protocol. The single time point evening measure inadvertently placed nearly half of the tests in the WMZ, thereby masking the underlying performance deficits and differences between lighting conditions [20]. Only when multiple points were measured, was there a clear effect of chronic variable sleep loss [32] and of lighting condition, reported herein. Compared to the SLS, we found that performance was better under DLS across bins 5 to 9, which correspond to a significant proportion of a typical working day (~11 am—5 pm). Performance at the beginning (bins 1 and 3) and end (bins 15 and 17, corresponding to the test timing of the once-per-day Cognition battery we previously reported on [20]) of the day were not different between lighting conditions, however. Therefore, contrary to performance evaluation using a single test per day [20], performance testing scheduled across the day enabled us to evaluate time awake effects and identify the full benefits of DLS on performance.

Although these data provide an important validation of the benefits of dynamic lighting in a real-world operational setting, the study has several limitations. For example, the limited sample size of each mission meant that we were likely underpowered to detect differences between the lighting conditions for all the PVT outcomes, despite the pattern of change being largely consistent across the different metrics (e.g. Figure 2). Even with the limited sample size; however, DLS lighting was significantly better than SLS lighting for minimizing attentional lapses (i.e. errors of omission or a failure to respond) which are arguably one of the more operationally relevant metrics measured by the PVT—i.e. the number of lapses is correlated to medical errors and [46] and driving performance [49]. Despite the correlation between PVT lapses and these operational outcomes, it could be argued that the PVT may not reflect performance on more difficult or important tasks [50] required during a space mission. The PVT is well-validated for reflecting both Process C and S, and the alerting effects of light [37, 38], and therefore represents a bellwether for general cognitive function. Nevertheless, future studies would benefit from adding additional measures of cognitive function (i.e. Cognition battery [21, 22]), including operational tasks, albeit with careful consideration of the timing of tests relative to the WMZ and other times of day where differences may be more difficult to detect (i.e. soon after waking due to sleep inertia). Additionally, although we measured and controlled for Process C in these analyses, we did not have a direct measure of Process S. Based on self-reported sleep diaries, we previously reported that sleep duration near-equaled the sleep opportunity (not surprising given the chronic sleep loss imposed) [20]; however, this method cannot provide information on sleep architecture, an important factor in determining underlying sleep loss due to Process S. Therefore, future studies should collect objective polysomnographic sleep data which would help to assess the time course of chronic variable sleep loss and light condition effects on sleep quality in addition to helping interpret the performance data [15]. Caffeine is a powerful stimulant that can mask performance impairment related to sleep and circadian misalignment [51], the lack of data on the crewmembers’ caffeine consumption habits, including potential differences between the lighting conditions or within individuals across different mission days (e.g. following 5- vs. 8-hour sleep opportunities), precluded the ability to control for this potential confound in our analyses. Future studies that allow caffeine should record the timing and amount of caffeine use; however, conducting these experiments entirely without caffeine would provide invaluable data on the cognitive and operational consequences of chronic variable sleep loss and lighting environment in the absence of the masking effects of a stimulant that may not be available on long-duration missions where stores of caffeine may be damaged or depleted as discussed in Rahman et al. [20].

Overall, these findings reinforce the need to evaluate performance several times throughout the day, not just once a day, when assessing the impact of countermeasures. Future missions should incorporate DLS to promote circadian alignment [20] and improve performance. In addition to deteriorating performance and safety, circadian misalignment and sleep loss are also associated with significant acute adverse physiological and metabolic effects [52–56] in the longer term, which may be important to consider during long-duration multi-year space missions. Preventing circadian misalignment and performance decrements due to sleep loss is therefore vital for the success of future space missions.

## Supplementary Material

zpae032_suppl_Supplementary_Material

## Data Availability

The datasets generated and analyzed during the current study are available by request in the NASA Life Sciences Data Archive: https://lsda.jsc.nasa.gov
