# Supplementary material for: The effect of a dynamic lighting schedule on neurobehavioral performance during a 45-day simulated space mission
Source: Sleep Adv. 2024 May 30;5(1):zpae032. doi: 10.1093/sleepadvances/zpae032 (PMC11187988; doi:10.1093/sleepadvances/zpae032)
Supplement: zpae032_suppl_Supplementary_Material [file zpae032_suppl_supplementary_material.pdf]

SUPPLEMENTAL MATERIAL

The effect of a dynamic lighting schedule on neurobehavioral performance during a 45-day simulated space mission

Leilah K. Grant,[1,2*] Brianne A. Kent,[1,2*] Shadab A. Rahman,[1,2] Melissa A. St. Hilaire[1,2], Crystal L. Kirkley,[3] Kevin B. Gregory,[3] Toni Clark,[4] John P. Hanifin,[5] Laura K. Barger[1,2], Charles A. Czeisler[1,2], George C. Brainard,[5] Steven W. Lockley,[1,2] Erin E. Flynn-Evans[3]

1. Division of Sleep and Circadian Disorders, Brigham & Women's Hospital, Boston, MA 02115
2. Division of Sleep Medicine, Harvard Medical School, Boston, MA 02115
3. NASA Ames Research Center, Fatigue Countermeasures Laboratory, Human Systems Integration Division, Moffett Field, CA, 94035
4. Johnson Space Center, Houston, TX 77058
5. Department of Neurology, Thomas Jefferson University, Philadelphia, PA 19107

* These authors contributed equally to this work

Correspondence should be address to:
Steven W Lockley, PhD, Division of Sleep and Circadian Disorders, Brigham & Women's Hospital, Boston, MA 02115, USA. E: slockley@hms.harvard.edu
Dr. Erin E. Flynn-Evans, Fatigue Countermeasures Laboratory N262-4, Human Systems Integration Division, NASA Ames Research Center, Moffett Field, CA, 94035, USA
E: erin.e.flynn-evans@nasa.gov

Light measurements across the HERA habitat for the SLS and DLS conditions

Light measurements were taken in the horizontal plane at a height of 72” for the following conditions and locations:

- SLS Level 1: Irradiance 1.47 ± 1.88 µW/cm^2; photon density 18.27 ± 0.60 log photons/cm^2/second; photopic lux 467 ± 614 lux; S-cone α-opic equivalent daylight index (EDI) lux 266 ± 333; M-cone α-opic EDI lux 409 ± 533; L-cone α-opic EDI lux 466 ± 612; rhodopic α-opic EDI lux 316 ± 400 lux; melanopic α-opic EDI lux 284 ± 354.

- SLS Level 2: irradiance 0.36 ± 0.19 µW/cm^2; photon density 17.95 ± 0.25 log photons/cm^2/second; photopic lux 108 ± 60 lux; S-cone α-opic equivalent daylight index (EDI) lux 61 ± 31; M-cone α-opic EDI lux 93 ± 51; L-cone α-opic EDI lux 109 ± 60; rhodopic α-opic EDI lux 69 ± 37 lux; melanopic α-opic EDI lux 62 ± 33.

- DLS Level 1 daytime setting: irradiance 3.97 ± 2.80 µW/cm^2; photon density 18.93 ± 0.34 log photons/cm^2/second; photopic lux 1210 ± 870 lux; S-cone α-opic equivalent daylight index (EDI) lux 1150 ± 807; M-cone α-opic EDI lux 1180 ± 845; L-cone α-opic EDI lux 1198 ± 857; rhodopic α-opic EDI lux 1099 ± 775 lux; melanopic α-opic EDI lux 1079 ± 748.

- DLS Level 1 evening/nighttime setting: irradiance 0.13 ± 0.14 µW/cm^2; photon density 17.41 ± 0.40 log photons/cm^2/second; photopic lux 44 ± 48 lux; S-cone α-opic equivalent daylight index (EDI) lux 15 ± 15; M-cone α-opic EDI lux 35 ± 38; L-cone α-opic EDI lux 44 ± 49; rhodopic α-opic EDI lux 25 ± 27 lux; melanopic α-opic EDI lux 21 ± 22.

- DLS Level 2 daytime setting: irradiance 0.34 ± 0.13 µW/cm^2; photon density 17.95 ± 0.16 log photons/cm^2/second; photopic lux 111 ± 44 lux; S-cone α-opic equivalent daylight index (EDI) lux 92 ± 38; M-cone α-opic EDI lux 103 ± 40; L-cone α-opic EDI lux 108 ± 42; rhodopic α-opic EDI lux 84 ± 32 lux; melanopic α-opic EDI lux 76 ± 28.

- DLS Level 2 evening setting: irradiance 0.01 ± 0.01 µW/cm^2; photon density 16.54 ± 0.31 log photons/cm^2/second; photopic lux 4 ± 3 lux; S-cone α-opic equivalent daylight index (EDI) lux 2 ± 1; M-cone α-opic EDI lux 4 ± 2; L-cone α-opic EDI lux 4 ± 3; rhodopic α-opic EDI lux 3 ± 2 lux; melanopic α-opic EDI lux 2 ± 1.

- DLS Level 2 nighttime setting: irradiance 0.01 ± 0.00 µW/cm^2; photon density 16.41 ± 0.25 log photons/cm^2/second; photopic lux 3 ± 1 lux; S-cone α-opic equivalent daylight index (EDI) lux 2 ± 1; M-cone α-opic EDI lux 2 ± 1; L-cone α-opic EDI lux 3 ± 1; rhodopic α-opic EDI lux 2 ± 1 lux; melanopic α-opic EDI lux 2 ± 1.

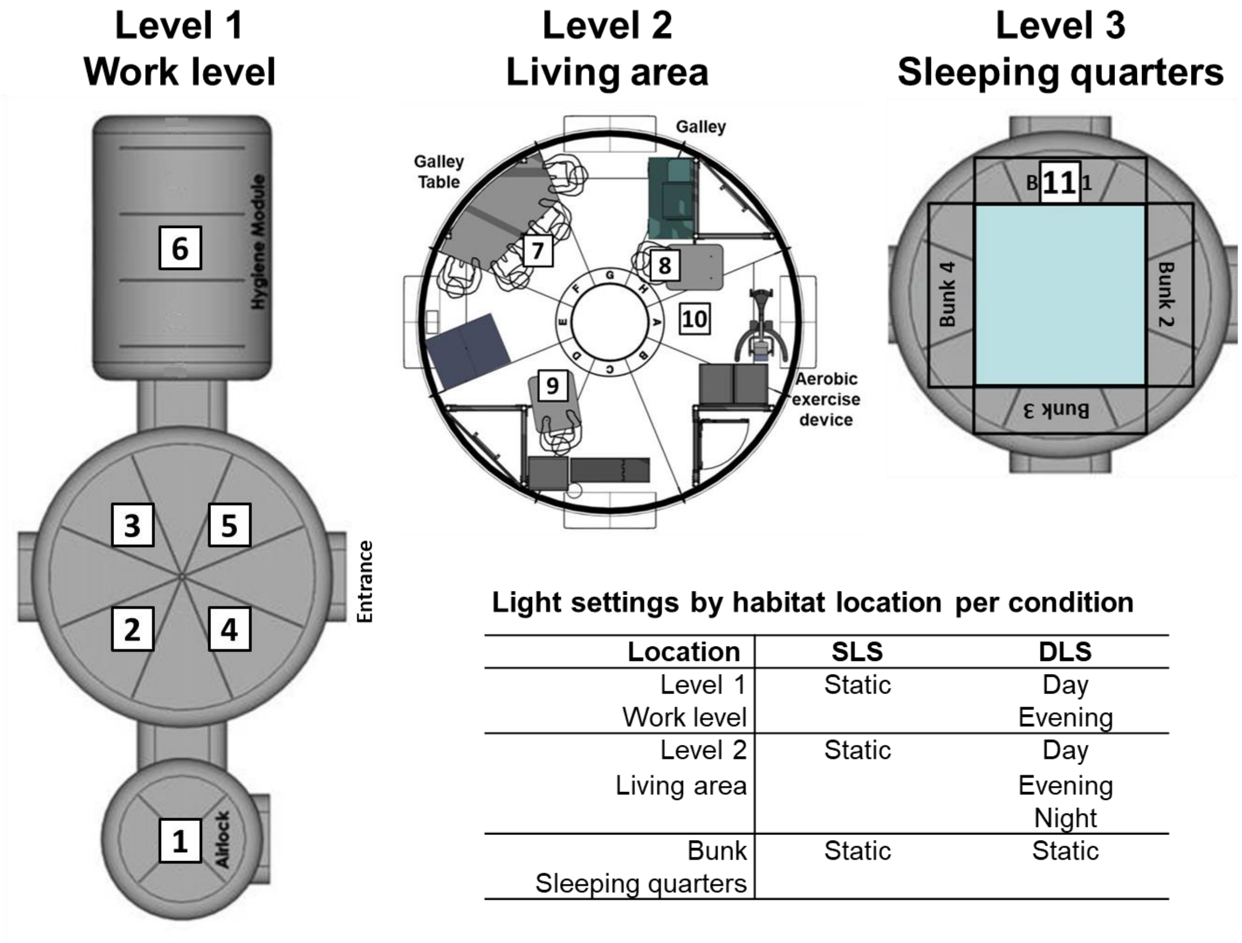

Light settings by habitat location per condition

Location	SLS	DLS
Level 1<br>Work level	Static	Day<br>Evening
Level 2<br>Living area	Static	Day<br>Evening<br>Night
Bunk<br>Sleeping quarters	Static	Static

Figure S1. HERA habitat floor plans for Levels 1, 2 and 3. The location of light readings in each level are denoted by a numbered box. The light settings available on each habitat level are listed in the table by lighting condition.

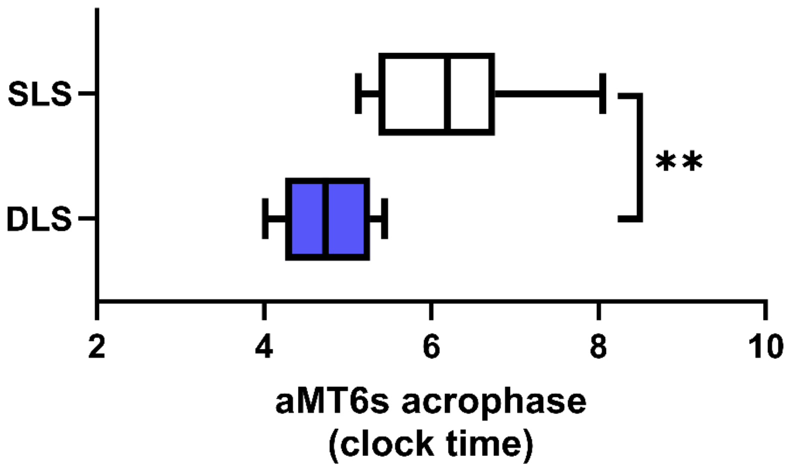

Figure S2. Acrophase of the urinary melatonin metabolite 6-sulphatoxymelatonin (aMT6s) by lighting condition (n=8 / condition). DLS = dynamic lighting schedule; SLS = standard lighting schedule; ** $p < 0.01$.

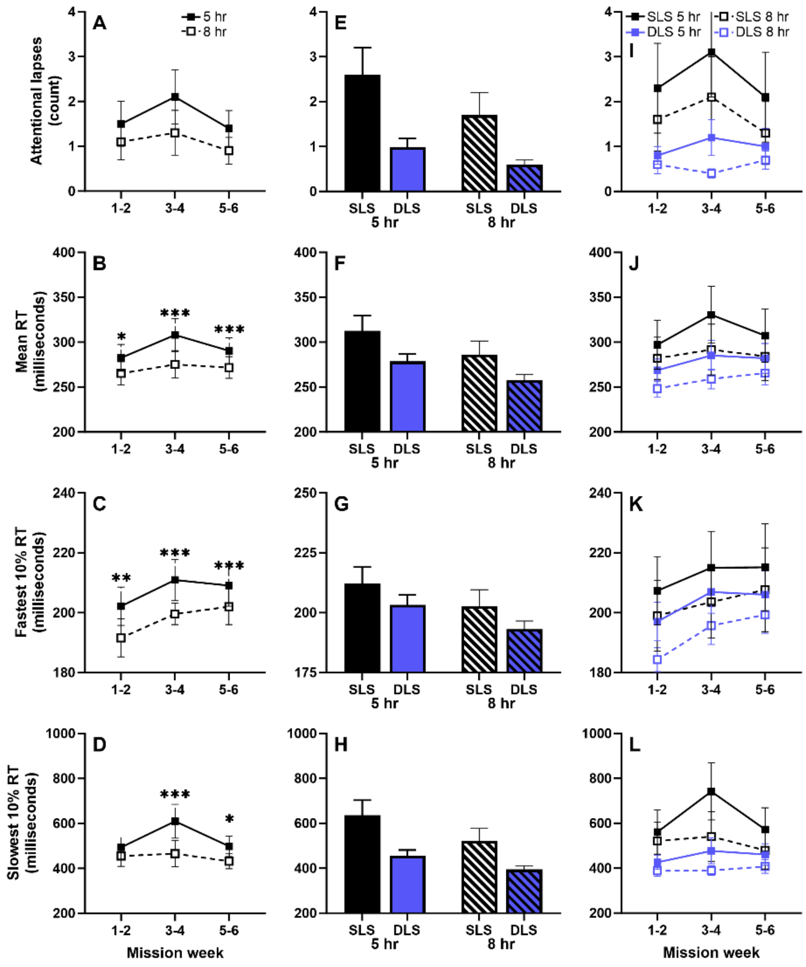

Figure S2. Interaction effects of light, sleep and time into mission on Psychomotor Vigilance Task (PVT) performance in 16 crewmembers (n=8 / lighting condition). Mean ± SEM of attentional lapses (top), mean reaction time (RT; center-top), 10% fastest RT (centre-bottom) and 10% slowest RT (bottom) are shown for the interaction between sleep condition and mission tertile (A-D), light condition by sleep condition (E-H) and light condition by sleep condition by mission tertile (I-L). Unadjusted data are plotted. DLS = dynamic lighting schedule; SLS = standard lighting schedule; * $p < 0.05$, ** $p < 0.01$, *** $p < 0.001$.
